# Induced Pluripotent Stem Cell-Derived Cardiac Progenitors Differentiate to Cardiomyocytes and Form Biosynthetic Tissues

**DOI:** 10.1371/journal.pone.0065963

**Published:** 2013-06-13

**Authors:** Nicolas Christoforou, Brian Liau, Syandan Chakraborty, Malathi Chellapan, Nenad Bursac, Kam W. Leong

**Affiliations:** Department of Biomedical Engineering, Duke University, Durham, North Carolina; Tokai University, Japan

## Abstract

The mammalian heart has little capacity to regenerate, and following injury the myocardium is replaced by non-contractile scar tissue. Consequently, increased wall stress and workload on the remaining myocardium leads to chamber dilation, dysfunction, and heart failure. Cell-based therapy with an autologous, epigenetically reprogrammed, and cardiac-committed progenitor cell source could potentially reverse this process by replacing the damaged myocardium with functional tissue. However, it is unclear whether cardiac progenitor cell-derived cardiomyocytes are capable of attaining levels of structural and functional maturity comparable to that of terminally-fated cardiomyocytes. Here, we first describe the derivation of mouse induced pluripotent stem (iPS) cells, which once differentiated allow for the enrichment of Nkx2-5(+) cardiac progenitors, and the cardiomyocyte-specific expression of the red fluorescent protein. We show that the cardiac progenitors are multipotent and capable of differentiating into endothelial cells, smooth muscle cells and cardiomyocytes. Moreover, cardiac progenitor selection corresponds to cKit(+) cell enrichment, while cardiomyocyte cell-lineage commitment is concomitant with dual expression of either cKit/Flk1 or cKit/Sca-1. We proceed to show that the cardiac progenitor-derived cardiomyocytes are capable of forming electrically and mechanically coupled large-scale 2D cell cultures with mature electrophysiological properties. Finally, we examine the cell progenitors’ ability to form electromechanically coherent macroscopic tissues, using a physiologically relevant 3D culture model and demonstrate that following long-term culture the cardiomyocytes align, and form robust electromechanical connections throughout the volume of the biosynthetic tissue construct. We conclude that the iPS cell-derived cardiac progenitors are a robust cell source for tissue engineering applications and a 3D culture platform for pharmacological screening and drug development studies.

## Introduction

An estimated one million US adults suffer a myocardial infarction annually, with six million having developed heart failure [1]. The calculated direct and indirect annual costs of managing heart disease are estimated at close to 180 billion dollars, amounting to approximately 9% of total healthcare expenditure in the US. Despite this significant health and economic burden, there are currently no effective treatments for heart failure short of a heart transplant. Drugs such as beta-blockers, angiotensin-converting enzyme inhibitors, and anti-arrhythmic medications manage the symptoms rather than tackle the underlying cause. Regenerative therapeutic approaches involving the transplantation of cardiogenic cells or engineered tissues can potentially address the latter by reducing post-infarct scar tissue formation [2], electromechanically integrating within the injured myocardium [3], and enhancing cardiac functional output [4], [5].

During mammalian development, the heart is the first organ to function in the embryo, and distinct populations of cardiac progenitors give rise to cells that populate the myocardium, including cardiomyocytes, smooth muscle and endothelial cells [6]. We and others, have previously described a unique population of embryonic stem (ES) cell-derived cardiac progenitor cells, which closely resemble their *in vivo* counterparts during developmental cardiogenesis both in gene expression and multipotent differentiation capacity [7]–[11]. In particular the ES cell-derived cardiac progenitors which are identified and isolated based on the expression of either T/Flk1 [8], Nkx2-5/Isl1/Flk1 [9], Nkx2-5/cKit [10], or Nkx2-5 [7], are capable of differentiating into cardiomyocytes, smooth muscle, and in certain cases endothelial cells. The physiological relevance of this *in vitro* differentiation system was demonstrated in screening assays which allowed the identification of novel genetic components active during the earliest stages of *in vivo* cardiogenesis [7], [12], [13]. Importantly, ES cell-derived cardiac progenitors are capable of engrafting in the infarcted myocardium, differentiating into the various cell lineages and effecting a significant functional improvement in cardiac output [14].

The recent discovery that mouse or human somatic cells can be epigenetically reprogrammed into induced pluripotent stem (iPS) cells closely resembling ES cells in their expanded proliferative capacity and differentiation potential has made it possible to derive immunocompatible genotype-specific and differentiated cell populations [15], [16]. Moreover, like their embryonic stem cell analogs, iPS cells retain the capacity to differentiate towards the cardiac cell lineage and form therapeutically relevant cells [17]–[19].

The potential success of a cardiac cell-based therapy depends 1) on the capacity of the therapeutic cell source to form cardiomyocytes that integrate electromechanically with the host myocardium and provide sufficient vascularization of the nascent tissue, 2) on the manner of cell delivery allowing for robust initial cell survival while ensuring long-term engraftment, differentiation, and functional integration, and 3) on the ability of donor cells to differentiate towards mature cardiomyocytes that are capable of reinforcing the failing heart without inducing life-threatening arrhythmias through electrophysiological incompatibility.

Although a range of cell types are being explored for therapeutic purposes, many ES cell or iPS cell-based therapeutic approaches hinge on the implantation of terminally fated cardiomyocytes [3], [20], [21]. However, the implantation of cardiomyocytes alone may not yield optimal results because vascular cells such as endothelial and smooth muscle cells are necessary for the formation of new vasculature to nourish the nascent muscle tissue [22], [23]. Thus the use of a multipotent cardiac progenitor cell population [7], [10], [14] may present a superior alternative. Although there is ample evidence that ES and iPS cell-derived cardiomyocytes mature temporally in terms of structural and functional parameters [24], [25], the maturation of cardiomyocytes derived from cardiac progenitors and their functional performance have not been examined in detail.

The assembly of an electromechanically functional 3D biosynthetic tissue is expected to provide a significantly improved therapeutic benefit compared to direct intracoronary or intramyocardial cell delivery, including efficient cell retention and survival at the site of injury, and prevention of ventricular remodeling by providing localized structural support. Additionally, a cardiac biosynthetic tissue generated from human cells would find utility in a microphysiological system for *in vitro* disease modeling and drug discovery [26]. Currently, pharmacological studies are commonly performed on either non-cardiac cell lines or in two-dimensional (monolayer) cultures of cardiomyocytes [27], [28]. To this end, we and others have recently demonstrated that cardiomyocyte maturation and function are significantly improved in three-dimensional biosynthetic tissues [29], [30]. Importantly, we discovered that although pure ES-derived cardiomyocytes alone were not capable of forming functional biosynthetic tissues and their formation necessitated addition of fibroblasts, ES-derived cardiac progenitor cells were an excellent single cell source for the assembly of functional 3D tissue constructs.

**Figure 1:**
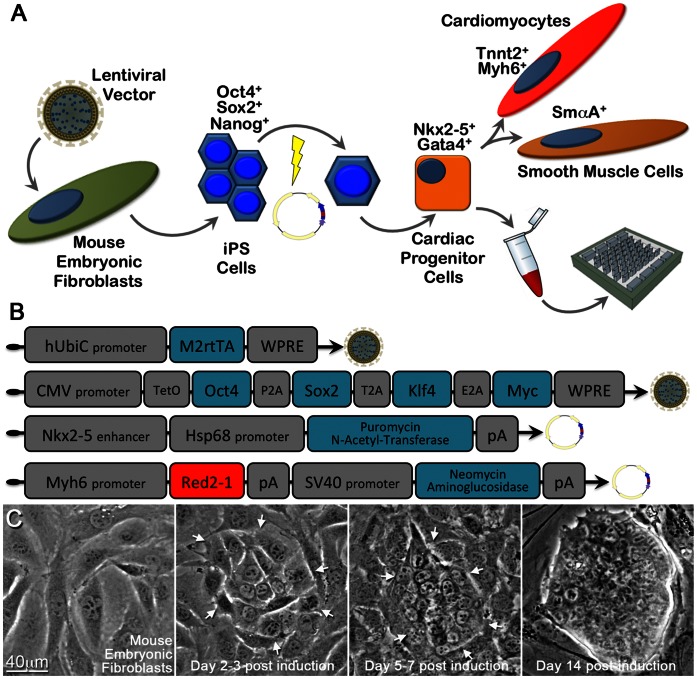
Experimental approach depiction and vectors utilized to derive iPS cells or cardiac progenitor cells. **A**. Mouse embryonic fibroblasts were epigenetically reprogrammed into induced pluripotent stem cells following their transduction with an inducible expression polycystronic lentiviral vector allowing the controlled expression of transcription factors Pou5f1, Sox2, Klf4, and Myc. Subsequently the derived iPS cells were stably transfected by electroporation with two DNA vectors allowing the genetic selection of cardiac progenitor cells (Nkx2-5 enhancer) and the detection of cardiomyocytes (Myh6 promoter). iPS cell-derived cardiac progenitors mainly differentiate into cardiomyocytes or smooth muscle and are used to assemble biosynthetic cardiac tissue constructs within hydrogel-based matrix and a polydimethylsiloxane mold. **B**. Schematic depiction of the lentiviral vectors used to derive iPS cells as well as the DNA vectors used to select the cardiac progenitors and identify cardiomyocytes. **C**. Brightfield images depicting epigenetic reprogramming of mouse embryonic fibroblasts into iPS cells. Clusters of fibroblasts with an increased proliferation rate and altered morphology ultimately becoming iPS cell colonies are indicated by white arrows.

We hypothesize that the generation of highly functional cardiac biosynthetic tissues using genotype-specific stem cell-derived cardiac progenitors would be valuable for both therapeutic interventions and drug development studies. In this study we aim to identify and characterize iPS cell-derived cardiac progenitor cells, determine their differentiation capacity, measure their functional phenotypic characteristics, and examine their capacity to form functional cardiac biosynthetic tissues. We derived and characterized iPS cells from mouse embryonic fibroblasts using a single polycistronic inducible expression lentiviral vector (Figure 1A) [15]. To generate cardiac progenitors, we derived iPS cells stably transfected with two DNA vectors: a *Nkx2-5* cardiac-specific enhancer element [7], [31] driving expression of puromycin N-acetyl transferase (PAC), enabling the isolation of pure populations of cardiac progenitor cells; and a *Myh6* promoter element [32] driving the expression of red fluorescent protein enabling the tracking of cardiac progenitor-descended cardiomyocytes. We utilized immunofluorescence, gene expression analysis, intracellular sharp microelectrode recordings and optical mapping of electrical propagation to assess the properties of differentiating cardiomyocytes. We also utilized a microfabrication-based approach [29] to guide the coalescence of the cardiac progenitors into functional engineered biosynthetic tissue constructs to determine their functional performance in a physiologically-relevant 3D setting.

## Materials and Methods

### Induced Pluripotent Stem Cell Derivation and Subsequent Transfection

Primary cultures of mouse embryonic fibroblasts (MEFs, Millipore) were epigenetically reprogrammed into iPS cells using a previously described polycystronic inducible-expression lentiviral vector [33] (FU.tet.on.OSKM). Briefly following cell transduction with the lentiviral vector, cells were cultured in stem cell culture medium (SC-Mdm) containing DMEM/High Glucose (Life Technologies), fetal bovine serum (15%, Atlanta Biologicals), non-essential amino acids (1%, Life Technologies), sodium pyruvate (1%, Life Technologies), Glutamax-I (1%, Life Technologies), βMercaptoethanol (55 µM, Life Technologies), gentamicin (25 µg/ml, Life Technologies), valproic acid (0.5 mM, Sigma), leukemia inhibitory factor or LIF (10^3 ^U/ml, ESGRO®, Millipore), and Doxycycline (10 µg/ml, Sigma). Doxycycline was removed 7 days post induction of transcription factor expression and individual iPS cell colonies were mechanically picked and expanded on a feeder layer of mitotically inactivated mouse embryonic fibroblasts.

Prior to the transfection process, iPS cells underwent two serial half-hour long pre-plating passages and subsequently cultured on gelatin-coated polystyrene for 24 hours in order to subtract the feeder layer cells. The next day the iPS cells were dissociated using Trypsin/EDTA (0.05%, Life Techologies) into a single cell suspension and counted. 5×10^6^ iPS cells were resuspended in 100 µl of ES cell nucleofection solution (VPH-1001) containing 8 µg of linearized Nkx2-5.Hsp68.PAC plasmid (FspI) and 2 µg of linearized Myh6.Red2.1.SV40.NeoR plasmid (SspI) and placed in an electroporation cuvette where the cells were electroporated using the A-023 nucleofection program (Nucleofector II device, Lonza Scientific). Transfected iPS cells were plated onto a fresh MEF feeder layer and neomycin selection (G418 sulfate, 100 µg/ml, Life Technologies) was initiated 2 days following nucleofection lasting until we detected clearly visible large undifferentiated iPS clonal colonies. Subsequently the colonies were mechanically picked, expanded as individual clones, and screened based on the described criteria.

### Production of Lentiviral Particles and DNA Plasmid Description

The DNA vector used for the production of lentiviral particles that allow the inducible expression of the four reprogramming transcription factors has been previously described (Addgene plasmid 20321, FU.tet.on.OSKM). To produce viral particles we used a previously described second generation lentivirus production system utilizing the psPAX2 (packaging proteins, Addgene plasmid 12660) and pMD2.G (envelope proteins, Addgene plasmid 12559) vectors (Dr. Didier Trono). We followed a procedure similar to the one described by Maherali et al [34]. Briefly HEK293T cells were cultured in a standard growth medium and maintained in T-75 tissue culture flasks pre-coated with a 0.1% solution of porcine gelatin (Sigma). Cells were allowed to reach 90% confluency at which point they were transfected in Opti-MEM® (Life Technologies) with a total of 24 µg of the three lentiviral vectors (12 µg FU.tet.on.OSKM, 7.7 µg of psPAX2 and 4.3 µg of pMD2.G) using Lipofectamine 2000 (Life Technologies). The supernatant containing the viral particles was collected at 48 and 96 hours following initial transfection with a final volume of 20 ml. The supernatant was subsequently concentrated to a final volume of approximately 300 µl using Amicon Ultra-15 centrifugal filter units (Millipore) and stored at 4°C for immediate use or in aliquots at −80°C for long-term use. To transduce primary MEFs, the cells were plated at a density of approximately 10,000 cells/cm^2^ in 6-well plates. The next day 1 ml of SC-Mdm medium containing 10 µl of each of the two viral concentrates and sequabrene (8 µg/ml, Sigma) was used to transduce the cells. One day following viral transduction the medium was exchanged with fresh SC-Mdm and the cells were subsequently passaged into a 10 cm cell culture plate for epigenetic reprogramming.

For the selection of iPS-derived cardiac progenitors we replaced the green fluorescent protein in the previously described Nkx2-5.Hsp68.eGFP DNA vector [7] with the puromycin N-acetyl-transferase gene (pORF39-PAC, Invivogen). To construct the Myh6.Red2-1.SV40.NeoR plasmid vector we cloned the Myh6 promoter element in the multi-cloning site of the pDsRed2-1 vector (Clontech).

### Maintenance and Differentiation of iPS Cells

Undifferentiated iPS cells were routinely maintained and passaged on a mitotically inactivated feeder layer of MEFs in SC-Mdm containing DMEM/High Glucose, 15% fetal bovine serum, non-essential amino acids, sodium pyruvate, Glutamax-I, βMercaptoethanol, Gentamicin, and LIF. To initiate differentiation 10^7^ iPS cells were suspended in 30 ml of differentiation medium (Diff-Mdm, same formula as SC-Mdm without LIF) and placed into 15 cm tissue culture plates pretreated with poly(2-hydroxyethyl methacrylate). Addition of ascorbic acid (100 µg/ml) was initiated on differentiation day 2 and approximately 90% of the Diff-Mdm was exchanged every 48 hours. For selection of iPS-derived cardiac progenitors, puromycin (5 µg/ml, Invivogen) was added to the Diff-Mdm on differentiation day 6 and kept in the medium for at least three days.

Cell aggregates comprised of iPS-derived cardiac progenitors were dissociated using a customized enzymatic solution containing Ca^2+^/Mg^2+^-free Hanks Buffered Salt Solution (HBSS, Life Technologies), Trypsin (2.5 mg/ml, Worthington Biochemicals), Collagenase Type I (2 mg/ml, Worthington Biochemicals), DNAse (1 mg/ml, Worthington Biochemicals), and EDTA (0.02%, Sigma). The aggregates were first washed twice with cold HBSS solution (4°C) containing only EDTA and then transferred in cold enzymatic solution in which they were incubated for 5–10 min. Subsequently the solution containing the aggregates was transferred in a 37°C water-bath where they were gently shaken for 10 min. Aggregates were then dissociated into a single cell suspension by gentle continuous pipetting and filtering through a 70 µm filter.

Confluent monolayer cultures of iPS-derived cardiac progenitors were prepared by plating 1500 cells/mm^2^ on either Aclar® coverslips (Electron Microscopy Sciences) or tissue culture polystyrene which was previously coated with a fibronectin solution (PBS, 25 µg/ml, Sigma). Following plating, iPS-derived cardiac progenitors were further cultured in Diff-Mdm for an additional 2 days and then transferred into a serum-free solution containing an equal ratio of DMEM/F12 and Neurobasal base medium N2 supplement (1×, Life Technologies), B27 supplement (1×, Life Technologies), Glutamax-I, non-essential amino acids, βMercaptoethanol, BSA (5 mg/ml, Sigma), and Gentamicin. Cultures of neonatal rat ventricular myocytes were prepared as previously described [35].

### Gene Expression Analysis

Primers were designed using NCBI primer-BLAST. In order to avoid polymerization of non-specific DNA amplimers, primers were required to span an exon-exon junction, and the primer pair was required to be separated by at least one intron on the corresponding genomic DNA (Table S1).

To perform qualitative RT.PCR analysis and ensure low level of DNA amplification without signal saturation, following total RNA isolation (Qiagen, 74104) a one step RT.PCR kit (Qiagen, 21212) was utilized with 23 cycles of amplification for Gapdh and 26–28 cycles (BioRad, MyCycler) for the rest of the genes analyzed. Quantitative RT.PCR analysis was performed on an ABI 7300 real-time thermocycler using the QuantiTect SYBR Green one-step RT.PCR kit (Qiagen, 204243). While performing the polymerase reactions on the real time thermocyclers we included the option to get a dissociation curve and also run the final reaction product on agarose gels in order to ensure that the only amplimers detected and measured were the expected ones. The SDS software (ABI, version 1.4) was used to analyze the raw data and then additional analysis was performed on Microsoft Excel. Relative quantification was performed using the ΔΔCt method. For each gene, one-way ANOVA was performed to determine if there were significant differences in gene expression throughout the experiment. The day of differentiation was used as the independent variable, and percentage gene expression (normalized against Day 14) as the dependent variable. In all cases, the p-value was found to be very small (<<0.01; not shown), indicating highly significant changes in gene expression. Holm-Bonferroni multiple comparison t-tests were then performed to determine whether individual data points were significantly different. Holm-corrected p-values <0.05 were deemed significantly different. All statistics were performed using the R software.

### Immunofluorescence, Image Capture and Analysis

Prior to initiating the immunostaining procedure cell monolayers, or biosynthetic tissue constructs were briefly washed with PBS and fixed using paraformaldehyde (2% in PBS, Electron Microscopy Sciences). Antibodies used included anti-Pou5f1 (Abcam, ab19857), anti-Fut4 (Millipore, MAB4301), anti-Actn2 (Sigma, A7732), anti-Tnnt2 (R&D Biosystems, MAB2444), anti-Myh6 (Abcam, ab15), anti-Myl2 (Abcam, ab48003), anti-Atp2a2 (Abcam, ab2861), anti-Acta2 (Abcam, ab5694), anti-Tagln (Abcam, ab10135), anti-Vwf (Abcam, ab6994), anti-Cdh2 (Abcam, ab12221), and anti-Gja1 (Santa Cruz, sc9059). Secondary antibodies for detection were purchased from Molecular Probes/Life Technologies and were all conjugated to the Alexa fluorochromes. Nuclei were detected using 4′, 6-diamidino-2-phenylindole, dihydrochloride (DAPI, Life Technologies). Fluorescent cell imaging was performed on either a Nikon Eclipse TE2000-U using a Roper Scientific CoolSnap HQ camera and the NIS Elements software suite or a Zeiss 510 inverted confocal microscope. Images analysis was performed using Nikon NIS Elements, Zeiss AIM LSM Image browser, BioView 3D, and Adobe Photoshop.

### Fluorescence-activated Cell Sorting

Embryoid bodies comprised of differentiating iPS cells were dissociated using serial gentle enzymatic dissociation (0.05% Trypsin, HBSS, 4 repeats) before or after addition of puromycin for selection of the Nkx2-5(+) cardiac progenitor cells. Cells were incubated with APC or PE/Cy7 conjugated antibodies or isotype controls and FACS was performed using a BD Biosciences FACSCanto instrument. Data analysis was performed on Flowing Software 2. All antibodies used for these experiments were purchased from Biolegend: APC anti-mouse c-kit (Cat # 105811), APC rat IgG2b isotype control (Cat # 400611), PE/cy7 anti-mouse Sca-1 (Cat # 108113), PE/cy7 rat IgG2a isotype control (Cat # 400521), and PE/cy7 anti-mouse Flk-1 (Cat # 136413).

### Microfabrication and Preparation of PDMS Molds

High-aspect ratio mesoscopic features were patterned onto silicon wafers using an optimized soft-lithography procedure. Specifically, ten cm diameter silicon wafers were first cleaned for 15 minutes in a 1∶3 mixture of hydrogen peroxide and concentrated sulfuric acid (Piranha Etch, 80°C). The silicon wafers were then washed in deionized water, dried with air and dehydrated on a hotplate at 200°C for 15 minutes. Upon cooling, a 250 µm thick layer of SU-8 100 photoresist (Microchem) was spun-coated onto the silicon wafer following the manufacturer’s protocol. The SU-8 coated wafer was then soft-baked at 95°C for 2 hours, followed by cooling to room temperature. Using this procedure, 5 additional 250 µm-thick layers were added to a final thickness of 1500 µm. The final soft-bake cycle was allowed to proceed at 95°C for additional 12 hours to promote evaporation of residual solvent from the SU-8 layers.

Photomasks were designed using Postscript and printed onto mylar transparencies (Advanced Reproductions). Photomasks were placed in contact with the soft-baked SU-8 layer while the areas outside the photomask boundaries were covered with aluminum foil to reduce the total surface area of SU-8 exposed to UV light, thereby reducing stress produced in the silicon wafer upon the subsequent hard-baking step. The SU-8 coated silicon wafers were then exposed to UV light (365 nm) at 12 mW cm^−2^ in a series of 12×1-minute exposures followed by 2-minute rest intervals so as to minimize heating of the photoresist layer. The exposed wafers were then hard-baked at 40°C for 48 hours to allow complete cross-linking of the exposed areas, followed by cooling to room temperature. Finally, the exposed wafers were developed overnight in polypropylene glycol monomethyl ether acetate (PGMEA) to dissolve unexposed SU-8, rinsed in isopropyl alcohol and dried using an air-gun.

SU-8 silicon templates were silanized overnight by placing them in a vacuum desiccator with approximately 500 µl of (tridecafluoro-1,1,2,2-tetrahydrooctyl)-1 trichlorosilane (henceforth referred to as ‘silane’). Sylgard 184 PDMS (Dow Corning) base was carefully poured over the SU-8 silicon template and allowed to cure overnight at 65°C, forming a negative impression of the SU-8 template (PDMS negative). The PDMS negative was detached from the SU-8 template, silanized and used as a template to create positive PDMS tissue mold which was used for the culture of tissue patches. PDMS tissue molds were sonicated in 70% ethanol for 1 hour, dried and subjected to a brief (1 minute) oxygen plasma ashing step, and kept in 70% ethanol. Nylon frames were pinned within the tissue molds to facilitate handling of the engineered tissues. Prior to cell culture, the molds were washed in 0.2% solution of F-127 pluronic (Invitrogen) in water for 1 hour to minimize hydrogel adhesion to the PDMS mold. PDMS molds were then dried with nitrogen and filled with previously prepared cell/hydrogel solution.

### Biosynthetic Tissue Assembly

Engineered cardiac tissues were fabricated using previously established protocols [29], [36]. In brief, iPS-derived cardiac progenitors were dissociated into a single cell suspension following two days of puromycin selection. The cells were resuspended in a mixture of fibrinogen (2 mg/ml, Sigma) containing Matrigel (10%, BD Biosciences). A small quantity of thrombin was added shortly before 120 µl of well-mixed cell suspension was pipetted into each microfabricated tissue mold and allowed to set for 45 minutes in a 37°C/5%CO_2_ incubator. Each engineered cardiac tissue patch was then immersed in 2 ml of serum-free medium containing 6-aminocaproic acid (1 mg/ml, Sigma). Engineered cardiac tissue patches were maintained for 14 days with media change every two days.

### Electrophysiological Characterization

22 mm circular Aclar® coverslips were coated with fibronectin as previously described [29] and placed in 12-well plates. Single-cell suspensions of iPS-derived cardiac progenitors were prepared by enzymatic dissociation and seeded onto the Aclar® coverslips at a density of 0.5 million cells per well in a 2 ml volume of Diff-Mdm. After 24 hours, medium was changed to serum-free which was used to maintain the monolayers for up to 16 days, with medium change every alternate day. Sharp microelectrode recordings were carried out using an Axon Instruments Multiclamp 700B setup with an ROE200 motorized headstage (Sutter Instruments). Sharp microelectrodes were pulled from borosilicate glass (Sutter Instruments BF100-50-10) using a Model P-97 brown-flaming micropipette puller (Sutter Instruments). Sharp electrodes were backfilled with 3 M KCl solution and had a resistance of 40–60 MΩ. Pipet capacitance neutralization of 2–5 µS was applied as appropriate to ensure accurate representation of upstroke velocity. To perform measurements, cardiac progenitor monolayer coverslips were placed in a heated chamber filled with Tyrodes solution.

Optical mapping of calcium transients was performed using our previously established protocol [37]. Briefly, engineered cardiac tissue patches were carefully removed from their molds and incubated for 30 minutes in the dark with 6 µM Rhod2-AM (Invitrogen) dissolved on Medium 199 (Gibco). Engineered tissue patches were then washed twice and incubated for a further 15 minutes in the dark with fresh Medium 199 to allow Rhod2-AM ester linkages to be completely cleaved. The engineered tissue patches were then transferred to a temperature-regulated chamber containing Tyrodes solution mounted on a standard Nikon fluorescence microscope. An image intensifier and 504-element photodiode array were positioned at the microscope's side-port. Point electrical stimulus was applied to the engineered tissue patch at 2 Hz using a bipolar platinum wire electrode, and calcium transients were imaged at 1000 Hz using a 4× objective and an excitation/emission of 560/600 nm.

## Results

### Derivation and Characterization of Induced Pluripotent Stem Cells

**Figure 2:**
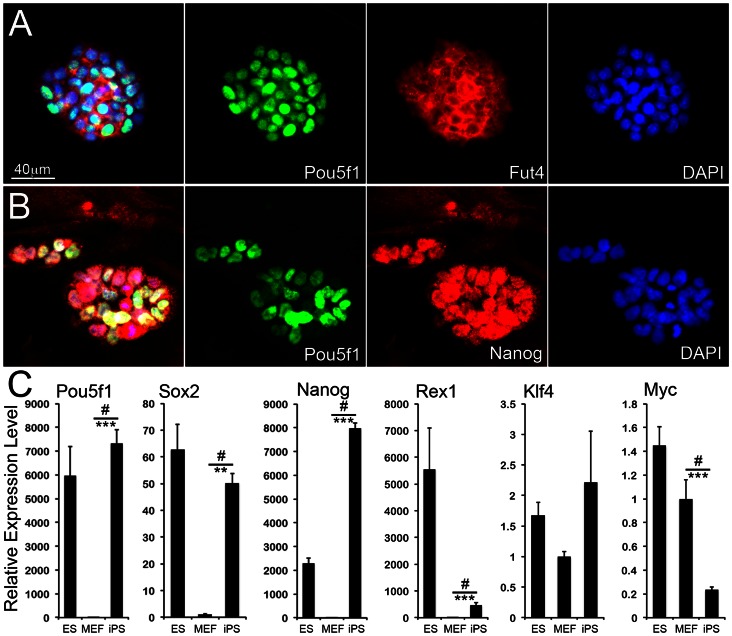
iPS cell characterization. **A–B**. Undifferentiated iPS cells express and co-localize transcription factors Pou5f1 and Nanog in their nuclei. Expression of the surface antigen Fut4 (SSEA-1) is also detected in Pou5f1-expressing iPS cells. Importantly the cells organize in tight compact colonies similarly to colonies of undifferentiated embryonic stem cells. **C**. Relative gene expression analysis for *Pou5f1*, *Sox2*, *Nanog*, *Rex1*, *Klf4*, and *Myc* following three serial expansion passages of the derived iPS cells in the absence of doxycycline. All gene expression levels are normalized against mouse embryonic fibroblasts using the ΔΔCt method. Error bars represent standard deviation. *, ** and *** indicate *p*<0.05, 0.01, 0.0001, respectively, and computed using one-tailed Student’s t-test for comparison of the gene expression levels measured for iPS cells with that measured for primary MEFs. ^#^indicates significant variance amongst all three groups as determined by one-way ANOVA analysis.

To epigenetically reprogram primary cultures of mouse embryonic fibroblasts (MEFs), we utilized a previously described [33] lentivirally-delivered polycistronic inducible-expression vector allowing the controlled over-expression of the murine transcription factors *Pou5f1* (*Oct4*), *Sox2*, *Klf4*, and *Myc* (Figure 1B). We readily detected clusters of cells with an increased proliferation rate and a higher nuclear-to-cytoplasmic size ratio as compared to non-transduced MEFs within two to three days following induction of transgene expression through the addition of doxycycline in the stem cell culture medium (SC-Mdm) containing valproic acid [38] (Figure 1C). The clusters of proliferating MEFs undergoing reprogramming grew larger temporally, and within 14 days post initiation of transcription factor over-expression we detected multi-layered compact iPS cell colonies with clearly marked borders closely resembling those of undifferentiated mouse ES cells [7]. Using immunofluorescence we determined that the generated iPS cells expressed Pou5f1 (Oct4, nuclear localization), Nanog (nuclear localization), and Fut4 (SSEA1, cell surface localization) (Figure 2A, B).

Individual iPS clonal colonies were subsequently mechanically picked, and expanded on mitotically-inactivated MEF feeder monolayers in the presence of SC-Mdm and without doxycycline. Following their initial clonal expansion (2–3 passages) we screened iPS cell clones using two selection criteria: firstly, we picked clones displaying a similar phenotype to that observed in cultures of undifferentiated ES cells; secondly, we selected clones with colonies that expressed the pluripotent stem cell proteins Pou5f1, Nanog, and Fut4 as determined by immunofluorescence. Using quantitative RT-PCR, we measured the relative gene expression level for *Pou5f1*, *Sox2*, *Nanog*, *Rex1*, *Klf4*, and *Myc* (Figure 2C, Figure S1). When measuring the gene expression level in iPS cells we detected a strong and highly significant increase in expression for *Pou5f1*, *Sox2*, *Nanog*, *Gdf3*, *Utf1*, and *Rex1* as compared to wild type MEFs. We did not detect a significant change in the expression level of *Klf4*, but observed a significant downregulation of *Myc*. Similarly to a previous report [39], we detected significant differences in the gene expression level of iPS cells as compared to undifferentiated ES cells (positive control). In particular we detected a significantly lower gene expression output for *Sox2*, *Myc*, and *Rex1*, a significantly higher expression for *Pou5f1*, and *Nanog*, and no significant differences for *Klf4*. Furthermore, by designing additional primers specific for a region outside the coding sequence of the reverse transcribed cDNA we were able to measure the relative expression level for the endogenous transcripts of *Pouf51*, *Sox2*, *Klf4*, and *Myc* while at the same time excluding the exogenous lentiviral transcripts (Figure S2C). Using the second set of primers we detected a pattern of expression similar to the one observed when using primers specific for the coding sequence. In particular, we detected a significant upregulation of endogenous *Pou5f1* and *Sox2* expression, a significant downregulation of *Myc* expression and no change for *Klf4* suggesting that the lentivirally-delivered exogenous transgenes were silenced following withdrawal of doxycycline and epigenetic reprogramming of MEFs into iPS cells.

### Induced Pluripotent Stem Cell-derived Cardiac Progenitor Cells

To selectively enrich for populations of iPS-derived cardiac progenitor cells, we utilized a genetic selection approach. Undifferentiated iPS cells were transfected by electroporation with two DNA plasmids to allow 1) the expression of puromycin N-acetyl-transferase (PAC) under the control of a previously described cardiac specific *Nkx2-5* enhancer element [7], [31], and 2) the expression of a red fluorescent protein (RFP) under the control of the *Myh6* promoter element [40] (Figure 1B). Following clonal selection and expansion of 48 stably transfected neomycin-resistant iPS clones we performed a screen for the cardiac-specific expression of the two transgenes. We readily identified iPS clones, which once differentiated produced spontaneously contracting RFP(+) cardiomyocytes (Movie S1). Moreover, the individual stably transfected clones retained expression of iPS markers as determined by immunofluorescence (Figure S2A, B).

**Figure 3:**
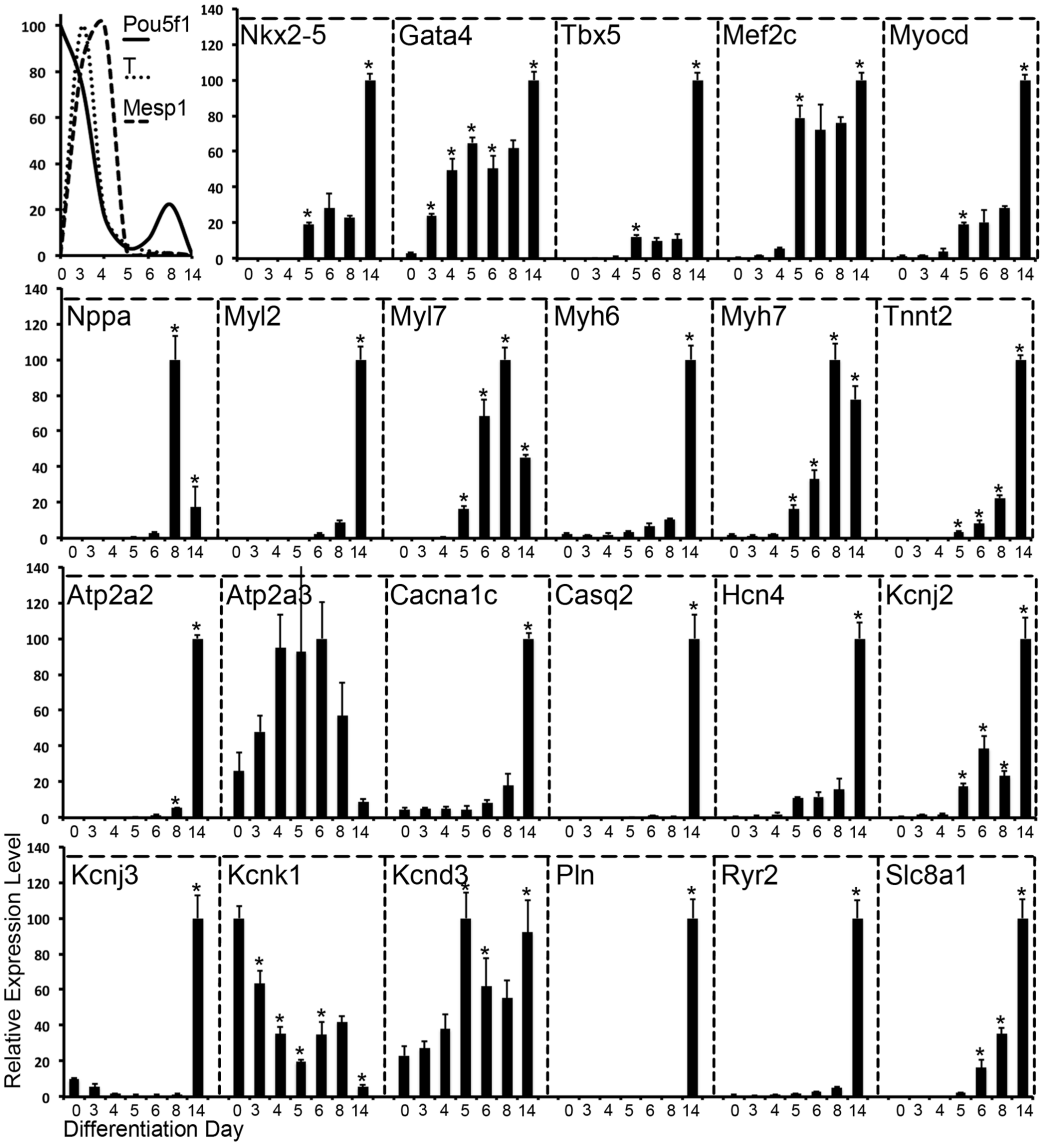
Gene expression analysis performed on differentiating mouse iPS cells. Temporal gene expression analysis was performed over the length of 14 days (7 timepoints) on cultures of differentiating mouse iPS cells: *Pou5f1* (undifferentiated iPS cells). *T*, and *Mesp1* (precardiac mesoderm). *Nkx2-5*, *Gata4*, *Tbx5*, *Mef2c*, and *Myocd* (early cardiac transcription factors). Nppa, Myl2, Myl7, Myh6, Myh7, and Tnnt2 (mature cardiomyocyte markers), *Atp2a2*, *Atp2a3*, *Cacna1c*, *Casq2*, *Hc4*, *Kcnj2*, *Kcnj3*, *Kcnk1*, *Kcnd3*, *Pln*, *Ryr2*, and *Slc8a1* (cardiomyocyte electrophysiology genes). All gene expression levels were normalized against day 0 undifferentiated iPS (except for *Pou5f1*) using the ΔΔCt method. Error bars represent standard deviation. Significant differences in gene expression were determined using one-way ANOVA. Independent variable: differentiation day, and dependent variable: percentage gene expression. In all cases, the p-value was found to be very small (<<0.01; not shown), indicating highly significant changes in gene expression. Holm-Bonferroni multiple comparison t-tests were then performed to determine whether an individual data point on a particular differentiation day was significantly different from that of the previous. Holm-corrected p-values <0.05 were deemed significantly different and denoted by an *.

To derive cardiac progenitor cells we used a modified version of our previously established differentiation protocol [7], [29]. In particular Nkx2-5(+) cardiac progenitors were enriched by adding puromycin to the culture medium as early as differentiation day 5. To examine the degree of cardiac progenitor induction and subsequent cell maturation in cultures of differentiating iPS cells, we performed temporal gene expression analysis. We screened for a range of genes expressed at different stages of cardiac development including pre-cardiac or cardiac transcription factors, and structural or electrophysiological genes (Figure 3, Figure S3). Following initiation of iPS differentiation we detected a rapid decrease in the relative expression level of *Pou5f1* followed by rapid upregulation of *Brachyury* (*T*, day 3) and *Mesp1* (day 4), indicative of nascent and pre-cardiac mesoderm induction [8], [41]. Initial expression of cardiac transcription factors *Nkx2-5*, *Tbx5*, *Mef2c*, and *Myocd* was detected on differentiation day 5 while initiation of *Gata4* expression was detected as early as day 3. Expression of contractile protein genes *Myl2*, *Myh6*, and *Tnnt2* was initiated between days 6 and 8 while their expression levels were strongly upregulated by day 14. Interestingly, the expression of *Myl7* and *Myh7* peaked on differentiation day 8 and subsequently decreased by day 14. These non-monotonic trends are characteristic of nascent cardiac differentiation where *Myl7* is expressed in all cardiomyocytes during early development, but becomes progressively more restricted to the atrial compartments as differentiation progresses [42]. *Myh7*, the ‘fetal’ form of cardiac myosin heavy chain, has been reported to decrease in expression relative to *Myh6* as cardiomyocytes undergo developmental hypertrophy [43]. Finally, we detected a late but very significant upregulation in the expression levels of *Atp2a2*, *Cacna1c*, *Casq2*, *Hcn4*, *Kcnj2*, *Kcnj3*, *Pln*, *Ryr2*, and *Slc8a1*, which are crucial for establishing a hyperpolarized resting membrane potential, as well as proper Na^+^, K^+^ and Ca^2+^ handling in the cell. We did not detect a significant change for *Atp2a3*, and *Kcnd3*, although we detected a decrease in the expression level of *Kcnk1*.

**Figure 4:**
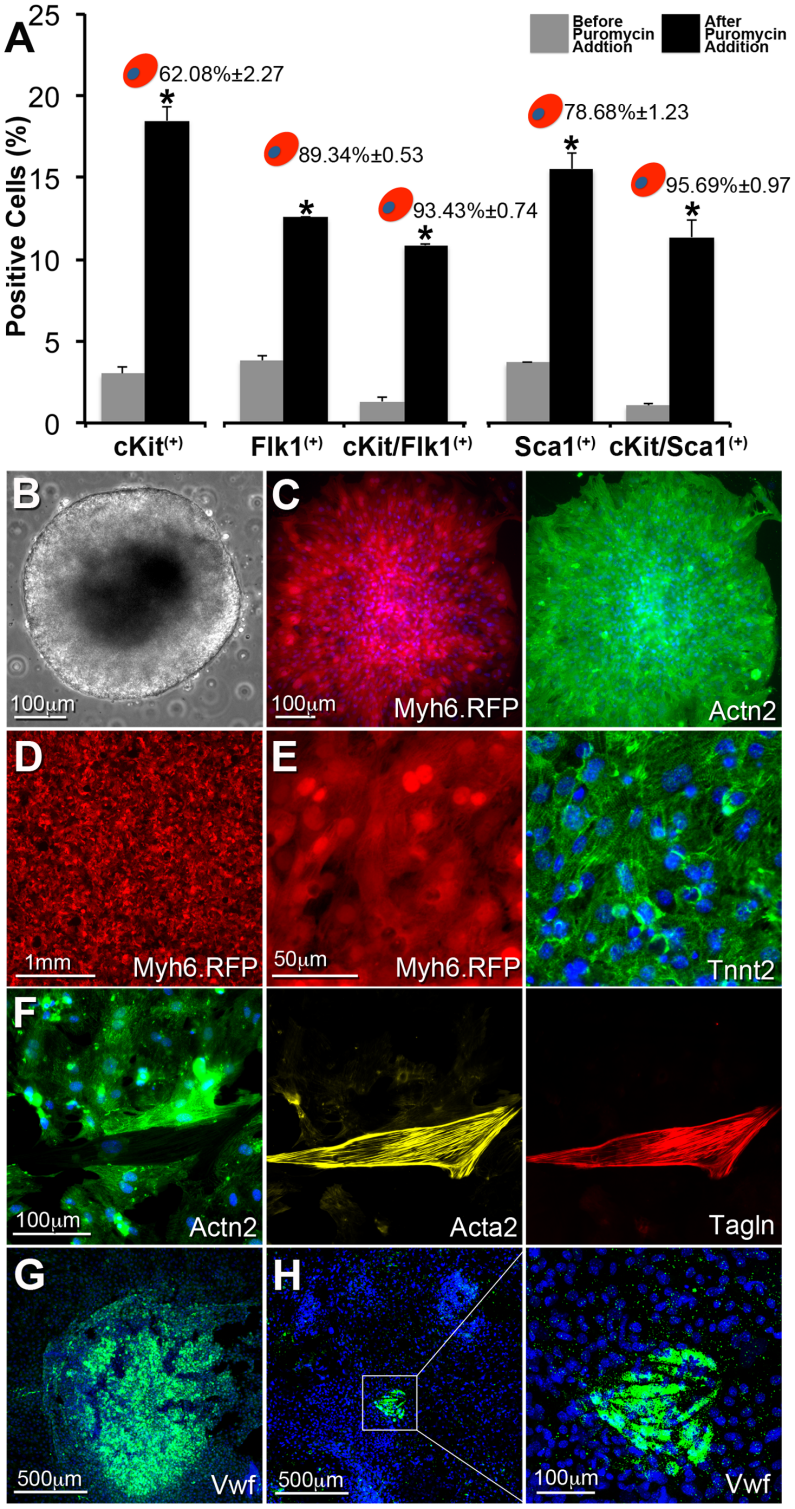
Cardiac progenitor characterization and multipotential differentiation capacity. **A**. The percentage of total cells expressing cell-surface antigens cKit, Flk1, and Sca-1 or the combinations of cKit/Flk1, and cKit/Sca-1 were determined by fluorescence-activated cell sorting before (gray) and after (black) puromycin addition for enriching Nkx2-5(+) cardiac progenitor cells. No RFP(+) cells were detected before addition of puromycin. The percentage of the five cell subpopulations also expressing RFP following puromycin addition is noted above the black columns. Error bars represent standard deviation. *denotes significant change (p<0.05) as determined by t-test statistical analysis. Isotype control antibodies were used as negative control in order to set the gates for the three cell surface antibodies. **B**. When cultured in suspension the derived iPS cells readily aggregated to form three-dimensional embryoid bodies which temporally differentiated into various cell types including spontaneously contracting cardiomyocytes detected as early as differentiation day 7. **C**. Following three days of puromycin antibiotic selection aggregates of spontaneously contracting RFP(+) cardiac progenitors were allowed to attach on gelatin-coated polystyrene. The cells in these aggregates stained positive for cardiac-specific actinin. **D**. Enzymatically dissociated and puromycin selected iPS-derived cardiac progenitors formed large-scale monolayers of spontaneously contracting cells. **E**. RFP-expressing (Myh6 promoter) and spontaneously contracting cardiomyocytes stained positive for the cardiac-specific marker Tnnt2. **F**. Acta2(+)/Tagln(+) smooth muscle cells were detected interspersed within the cultures of Act2(+) cardiomyocytes. **G–H**. Rare colonies of endothelial cells with varying size were detected within the cell monolayers as determined by immunostaining for Vwf.

We have previously described the identification of a multipotent population of ES cell-derived cardiac progenitors based on the activation of a cardiac-specific enhancer element of the Nkx2-5 transcription factor [7]. Similar reports have also established that the Nkx2-5 progenitors additionally express the cKit [10] or Flk1 [9] cell surface markers. We set out to determine whether the iPS cell-derived Nkx2-5(+) cardiac progenitors also expressed cKit, Flk1, or Sca-1 which has been previously reported to be expressed on adult cardiac stem cells [44], [45] (Figure 4A). During the early differentiation process, and prior to the addition of puromycin for enrichment of Nkx2-5(+) cardiac progenitors, we detected a small population of single cKit (3.00±0.39%) positive cells, while not detecting a significant population of double positive cells for either cKit/Flk1 (1.31±0.27) or cKit/Sca-1 (1.05±0.12). Also, no RFP(+) cells were detected at the early differentiation timepoint, which is concomitant with the absence of Myh6 transcripts as determined by gene expression analysis. Following a short-term enrichment for the Nkx2-5(+) cardiac progenitors we detected a significant increase in the population of cells expressing cKit (18.47±0.84%). We also detected RFP expression suggesting initiation of the cardiomyocyte lineage commitment in a subpopulation of the enriched cardiac progenitors (16.00±0.93%). We examined whether expression of the cell surface markers correlated with RFP expression and thus cardiomyocyte lineage commitment. We first determined that approximately two thirds (62.08±2.27%) of the cKit(+) cells also expressed RFP. Interestingly, dual expression of either cKit/Flk1 or cKit/Sca-1 tightly correlated with RFP expression (93.43±0.74% and 95.69±0.97% respectively), indicating that co-expression of either Flk1 or Sca-1 with cKit and Nkx2-5(+) is associated with cardiomyocyte-lineage commitment in the cardiac progenitor cell population.

Phenotypically when cultured in suspension the derived and stably transfected iPS cells readily aggregated, forming 3D embryoid bodies (Figure 4B), which increased in size temporally. Addition of puromycin in the culture medium starting on differentiation day 5 or 6 induced short-term large-scale cell death while a subpopulation of cells remained alive in aggregates. Expression of RFP was initially detected between days 6 and 7 and found in aggregates of spontaneously contracting cardiomyocytes that also expressed Actn2(+) (Figure 4C). We developed a customized enzymatic blend to dissociate the cell aggregates into a single cell suspension while ensuring high cell viability and a low level of post-dissociation cell aggregation. The dissociated single cells readily attached on fibronectin-coated polystyrene or glass coverslips forming large-scale monolayers (>10 cm^2^) of synchronously contracting cells indicative of potential intercellular electrical coupling, and maintained their structural integrity over a 4-week period (Figure 4D–E, Movie S2). To examine the multi-lineage differentiation potential of the iPS-derived cardiac progenitors, following long-term culture we performed immunofluorescence on cell monolayers. Although the majority of cardiac progenitors differentiated into cardiomyocytes as determined by Actn2 staining, we readily detected smooth muscle cells that stained double positive for Acta2 and Tagln (Figure 4F). In total, 10.99±5.18% of the cell population stained positive for the smooth muscle markers. Finally, we determined that the cardiac progenitors also differentiated into the endothelial cell lineage. In particular we detected both large and small colonies of cells staining positive for Von Willebrand factor (Figure 4G, H) [7].

**Figure 5:**
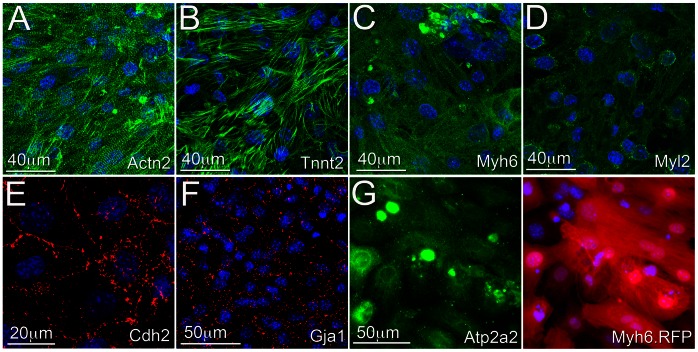
Immunofluorescence characterization of cardiomyocytes differentiated from the cardiac progenitor cells. **A–D**. The cardiomyocytes formed well-defined cross-striated sarcomeric structures as determined by the expression and spatial organization of Actn2, Tnnt2, and Myh6 and also expressed the ventricular specific protein Myl2. **E–F**. The cells also formed robust intercellular electrical and mechanical connections as determined by the spatial organization of Cdh2 and Gja1. **G**. RFP(+) cardiomyocytes stained positive for sodium/potassium ATPase (Atp2a2).

We also determined that the iPS-derived cardiac progenitors differentiated into relatively mature cardiomyocytes as demonstrated by the level of sarcomeric organization of proteins Actn2, Tnnt2, and Myh6 (Figure 5A–C), and expression of the ventricular Myl2 protein (Figure 5D). Furthermore, cardiomyocytes in monolayers exhibited intercellular coupling via the expression and spatial organization of Cdh2 (N-Cadherin) and Gja1 (Connexin 43), indicative of the formation of functional electromechanical connections (Figure 5E–F) and also expressed the cardiac specific ATPase Atp2a2 (Figure 5G). Isolated cultures of neonatal rat ventricular myocytes were used as a positive control cell source in order to validate the specificity of antibodies used to characterize iPS-derived cardiac progenitors and their differentiated progeny (Figure S4).

### Electrophysiological Characterization of iPS-derived Cardiac Progenitors

**Figure 6:**
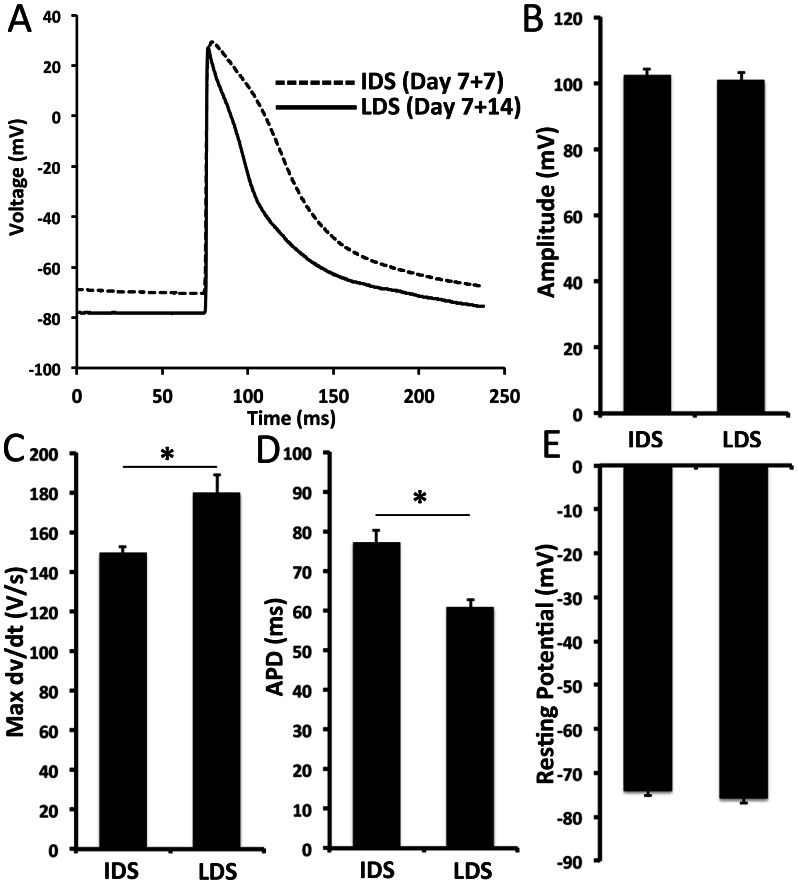
Intracellular microelectrode characterization of iPS cell-derived cardiomyocytes. The electrophysiology of RFP-expressing cardiomyocytes matured temporally in culture. **A**. Representative cardiomyocyte action potential traces at the intermediate differentiation stage (IDS, Day 7+7) or late differentiation stage (LDS, Day 7+14). Between IDS and LDS in culture, **B**. Action potential amplitude was not significantly different (102.4±1.9 mV versus 100.9±2.2 mV). **C**. Maximum action potential upstroke velocity increased from 149.3±3.5V/s to 179.5±9.3V/s. **D**. Action potential duration at 80% repolarization decreased from 77.2±3.2 ms to 60.9±1.7 ms. **E**. Maximum diastolic potential was not significantly different (−74.1±1.1 mV versus −75.9±1.1 mV).

Sharp electrode recordings in differentiating cardiac progenitor monolayers were performed either at an intermediate differentiation stage (IDS, Differentiation day 7+7), or at a late differentiation stage (LDS, Differentiation day 7+14) (Figure 6A). At both IDS and LDS, the cells exhibited synchronous spontaneous beating at 1–2 Hz. Moreover, at both IDS and LDS, the differentiating cardiac progenitors exhibited similarly low, stable resting potentials of −74.12±1.07 mV and −75.86±1.1 mV respectively (Figure 6E, p>0.05), as well as similar action potential amplitudes of 95.4±2.4 mV and 99.1±1.4 mV respectively (Figure 6B, p<0.05). Action potential upstroke velocity at IDS (149.3±3.6V/s) was found to be significantly slower than at LDS (179.5±9.3V/s; p<0.05) (Figure 6C). APD_80_ at IDS (77.2±3.2 ms) was significantly prolonged as compared to LDS (60.9±1.7 ms) (Figure 6D). Taken together, the cardiac progenitor cell-derived cardiomyocytes electrically matured in monolayer cultures, consistent with previous reports involving mouse ES and iPS cell-derived cardiomyocytes [24], [46], [47].

### Engineering and Characterization of Biosynthetic Cardiac Tissues

**Figure 7:**
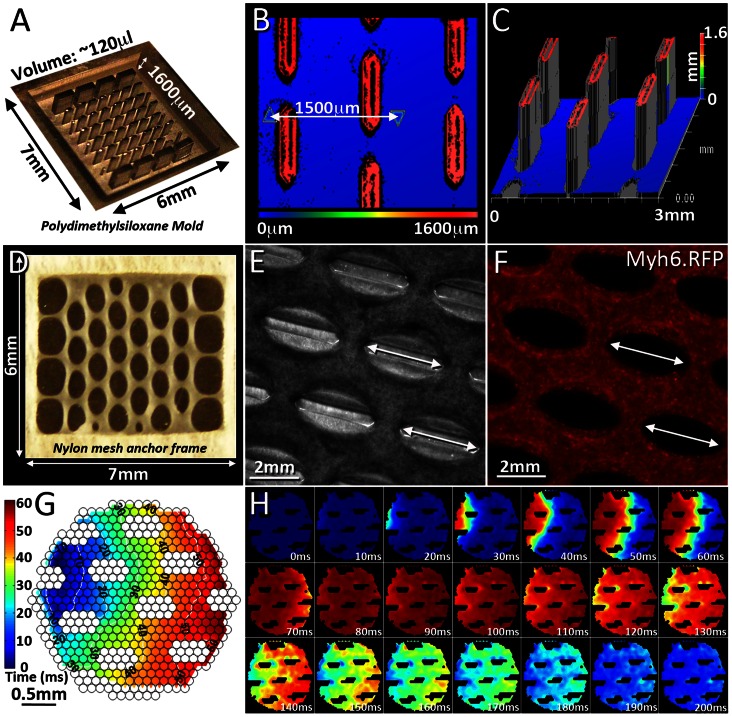
Engineered tissue patches and optical mapping of action potential propagation. Microfabricated tissue molds cast in PDMS were used to direct the alignment of iPS cell-derived cardiac progenitor cells in three-dimensional biosynthetic tissue constructs. **A**. PDMS tissue mold (sputtered with chrome for greater contrast). **B–C.** Zygometer surface profiles of the PDMS mold showing 1600 µm tall hexagonal features. Individual hexagons are 800 µm in length and 200 µm in width. **D.** An engineered tissue patch removed from its mold, bordered by a nylon mesh frame to facilitate handling. Elliptical void spaces are a result of tissue compaction away from the hexagonal features. **E–F.** Live brightfield and RFP images of the engineered tissue construct within its mold, showing tissue compaction forming elliptical pores around the hexagonal features and live cardiomyocytes within the tissue patch. **G.** Isochrone map showing activation times across the tissue patch during propagation (left to right) of an action potential. Blue pixels represent sites of early activation, and red pixels represent sites of late activation. **H.** False-color images representing a calcium transient traversing a tissue patch from left to right as a result of action potential firing. Red pixels represent a high intracellular calcium concentration, and blue pixels represent a low concentration.

To assemble the three-dimensional engineered tissues we first utilized a recently described [29] soft-lithography process to fabricate high-aspect ratio mesoscopic features (posts) patterned onto silicon wafers (Figure 7A–C). Subsequently, the microfabricated molds were recreated with polydimethylsiloxane (PDMS) by double-casting. Following two days of antibiotic selection (differentiation days 6–7) iPS-derived cardiac progenitors were enzymatically dissociated into single cell suspensions, mixed within a fibrin/matrigel hydrogel, and applied into the PDMS molds. To ensure a high level of initial cell viability while avoiding proliferation of any remaining undifferentiated iPS cells we cultured the newly formed tissues in high serum medium in the presence of puromycin for an additional two days and subsequently transferred them in serum-free culture conditions.

Within three to four days after gelation, we detected initiation of hydrogel compaction (Figure 7D) and observed spontaneous contractile activity. The amplitude of spontaneous contractions increased correlating with the detection of an increasing number of RFP(+) cardiomyocytes that became elongated, particularly along the curved hydrogel edges which formed around the PDMS posts (Figure 7E–F, Movies S3–S6). Finally within two weeks of culture in serum free conditions, we detected unidirectional cardiomyocyte alignment within tissue constructs (Movie S7) accompanied with macroscopic contractile activity.

To functionally characterize the biosynthetic tissue constructs we first performed optical mapping of intracellular calcium transients using our previously established protocols [37]. We determined that when electrically stimulated by point electrode, tissue constructs sustained macroscopically uniform action potential propagation with conduction velocities between 5.0–7.7 cm/s (n = 3, Figure 7G–H, Movie S8).

**Figure 8:**
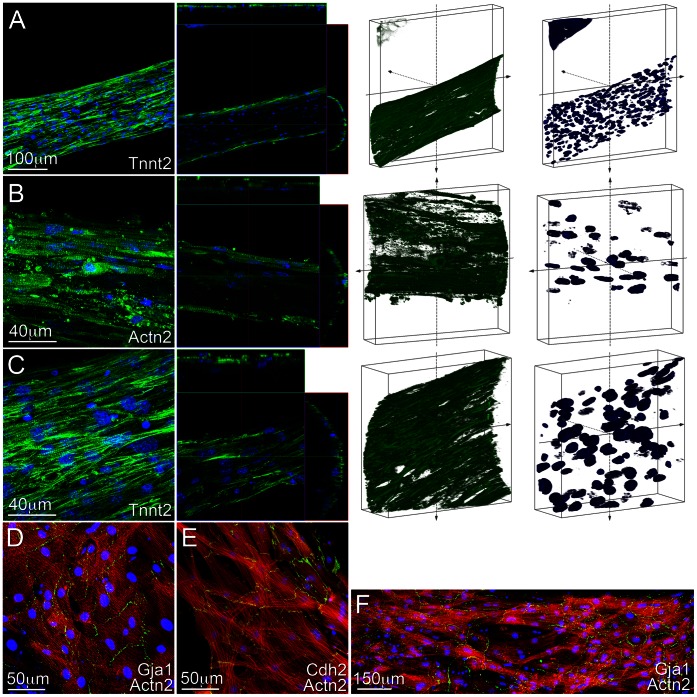
Confocal microscopy of three-dimensional engineered biosynthetic tissue constructs containing iPS cell-derived differentiated cardiac progenitor cells. Representative images of the immunostained cells within the tissue constructs. This series of images was taken from an area located between the large and small pores within the construct. **A.** Cells within the biosynthetic tissue constructs significantly compacted the original hydrogel matrix, allowing for the formation of high-density intertwined string-like fibers. The cardiomyocyte cytoskeleton was elongated and aligned along the long-axis of the biosynthetic tissue constructs. **B–C.** The cytoskeletal sarcomeric arrangement was highly organized and placed perpendicular to the long axis of the biosynthetic tissues. **D–F**. The cardiomyocytes formed robust electromechanical connections (gap and adherens junctions) as determined by the level of expression and spatial organization of Gja1 and Cdh2 over long distances. These connections were often detected perpendicular to the long-axis of the tissue constructs.

Finally we examined the engineered tissue structure with respect to the spatial distribution of cells and their interconnectivity. We observed that the maturing cells had significantly compacted the original hydrogel matrix, allowing for the formation of high-density intertwined string-like unidirectional fibers with a width ranging between 150 and 500 µm (Figure 8A). The cardiomyocyte cytoskeleton was elongated and aligned along the long-axis of the biosynthetic tissue constructs. Interestingly the cardiomyocytes remained on the external surface of each individual fiber, suggesting that inadequate nutrient transport within the entire volume of the hydrogel was prohibitory for thick tissue formation. The cytoskeleton of individual cardiomyocytes was comprised of highly organized mature sarcomeric structures which ran perpendicular to the long axis of the biosynthetic tissues as determined by staining for either Actn2 or Tnnt2 (Figure 8B–C). To examine whether the cardiomyocytes were electromechanically coupled we stained the cells with antibodies against Cdh2 and Gja1, markers for the mechanical adherens junctions and electrical gap junctions, respectively (Figure 8D–E). We determined that the cells formed robust electrical and mechanical connections throughout the entire tissue, indicating a high degree of structural integrity. Importantly this level of electrical coupling was consistent along large-scale regions of the biosynthetic tissue constructs (Figure 8F).

## Discussion

Recent studies set out to establish a correlation between the distinct cell populations present early during development or ES-cell differentiation in pre-cardiac or cardiac mesoderm and their lineage-commitment into cardiomyocytes, smooth muscle cells, and endothelial cells [8]–[10]. *Kattman et al.* first reported that a late expression of Flk1 in conjunction with the pre-cardiac mesoderm marker Brachyury selectively mark tripotent cardiac progenitor cells which express Nkx2-5 and are capable of differentiating into smooth muscle, endothelial cells, and cardiomyocytes [8]. *Wu et al.* also reported that Nkx2-5(+) cardiac progenitors express cKit and can differentiate into smooth muscle and cardiomyocytes [10]. Finally, *Moretti et al.* reported that Isl1/Nkx2-5(+) cardiac progenitors express Flk1 and are also tripotent in their differentiation capacity [9]. Here, we utilize a two plasmid system allowing us to selectively enrich for iPS-derived Nkx2-5(+) cardiac progenitors and follow their commitment towards the cardiomyocyte cell lineage through expression of RFP. We first demonstrate that the cardiac progenitors are tripotent and capable of differentiating into the three cell lineages with the myocyte cell lineage being the most prevalent. We show that Nkx2-5(+) cell selection positively correlates with cKit enrichment although only approximately two thirds of these cells commit towards the myocyte cell lineage. Interestingly, dual expression of cKit with either Flk1 or Sca-1 highly correlates with RFP expression in the Nkx2-5(+) progenitor population, suggesting that acquisition of either of the two markers by the Nkx2-5/cKit cells predicts their commitment towards the myocyte lineage. In the future we plan to examine whether endothelial cell-lineage commitment within the Nkx2-5(+) cardiac progenitor cell population can be predicted based on the cell-surface signature of cKit(+)/Flk1(−) or cKit(−)/Flk1(+).

Successful reprogramming of mouse embryonic fibroblasts into iPS cells involves incremental levels of characterization, including mesenchymal-to-epithelial transition and acquisition of an ES cell-like phenotype, activation of a combination of endogenous genes associated with pluripotency, e.g. Pou5f1, Sox2, and Nanog, expression of the appropriate cell-surface markers, e.g. Ssea1, multipotent differentiation capacity into cell types representing the three germ layers, capacity to form *in vivo* teratomas, and ultimately viable animal production through tetraploid complementation [16], [48]. Although here we perform a detailed characterization of the pluripotent phenotype of the iPS cells derived for the purpose of this study, one limitation is that we have not assayed their ability to form teratomas or transgenic animals as we are particularly interested in their ability to differentiate into the cardiovascular cell lineages, which we clearly demonstrate.

Cardiac progenitor cells have been previously isolated from differentiating ES cells using various strategies including specific cell-surface marker expression, e.g. Flk-1/KDR [8], [49], or through the activation of particular promoter or enhancer elements, e.g. Mesp1 [50] and Isl-1 [9], [51]. These progenitors represent a dynamic state during ES cell differentiation, and for murine cells are typically isolated after differentiation day 5 when transcripts coding for early cardiac transcription factors are first detected [10], [29], [49], [51].

During embryonic development or pluripotent stem cell differentiation, as the cardiac progenitors become committed and differentiate towards the myocyte cell lineage, the resulting cells are relatively immature and express significantly lower levels of cardiac-specific genes such as *Myh6*, *Myl2* and *Slc8a1*. In contrast, ES cell-derived cardiomyocytes isolated based on the activation of either the Myh6 [32], Myl2 [52] or Slc8a1 [53] promoter element have been shown to be more mature. In particular these cardiomyocytes express high levels of promoter-associated genes and show characteristic electrophysiological and structural features, such as hyperpolarized and stable resting membrane potentials, fast maximum upstroke velocity during action potential depolarization, and well-formed myosin heavy chain, actinin and troponin sarcomeres.

The capacity of the relatively immature cardiac progenitors to reach a fully functional and mature cardiomyocyte phenotype following their enrichment and in the absence of a paracrine cardio-inducing niche has come into question. *Kim et al.* observed that the presence of non-cardiomyocytes in differentiating embryoid bodies influenced the ability of nascent human ES cell-derived cardiomyocytes to reach full electrophysiological maturity [25]. In particular cardiomyocytes isolated from their supporting non-cardiomyocytes at an early stage of differentiation maintained a more depolarized resting membrane potential and a slower action potential upstroke velocity, whereas cardiomyocytes allowed to differentiate in the presence of supporting non-cardiomyocytes showed a more hyperpolarized resting membrane potential and a faster upstroke velocity. Similarly, *Arai et al.* observed that signaling between visceral endodermal cells and nascent cardiomyocytes was essential for functional cardiac differentiation [54]. In the absence of such signaling cues, even though cardiac progenitors upregulated expression of cardiac markers such as *Myh6*, they failed to assume a spontaneously contracting phenotype. The findings of these studies suggest that ES cell-derived myocyte-lineage committed cardiac progenitors may require additional paracrine signaling to reach a fully mature cardiomyocyte phenotype.

It has been hypothesized that ES cell- or iPS cell-derived cardiac progenitor cells are an ideal cell source for cell-based therapies because their multipotent phenotype would allow them to generate new functional cardiomyocytes and establish the necessary vasculature. If, however, early isolation of these cells would negatively impact their potential to form fully mature and functional cardiomyocytes then their utilization for cell-based therapies would be compromised.

Here we address this question by demonstrating that Nkx2-5(+) cardiac progenitor cell-derived cardiomyocytes have robust expression of structural markers which are characteristic of highly differentiated cardiomyocytes i.e. highly organized Actn2, Tnnt2 and Myh6 cross-striated sarcomeres. They also express high levels of Kcnj2 and Kcnj3, which allow a more hyperpolarized resting membrane potential characteristic of mature cardiomyocytes [55]. This was corroborated by our intracellular sharp electrode experiments where we measured hyperpolarized, stable resting membrane potentials between −74 mV and −76 mV (Fig. 5D), in good agreement with resting potential of terminally differentiated mouse cardiomyocytes [56], [57]. Furthermore, over the time course of cardiac progenitor cell differentiation, we measured a significant increase in the maximum upstroke velocity without significant changes in resting membrane potential, suggestive of an increase in fast sodium current density (Fig. 5B), a hallmark of functional cardiac differentiation. The observed shortening of APD_80_ over time is likely attributed to an increase in I_to_, the main repolarizing current in murine cardiomyocytes. Notably, these mature electrophysiological properties are consistent with those measured in terminally differentiated ES cell-derived cardiomyocytes [24] and Myl2-selected cardiomyocytes [52]. Specifically, *Maltsev et al.* [24] observed stable resting potentials of −74±5.1 mV in late-stage, terminally differentiated ES cell-derived cardiomyocytes, with an average upstroke velocity of 232±62 V/s and an average action potential duration ranging from 124–148 ms. *Muller et al.* [52] observed stable resting potentials of −68.6±2.8 mV and an average action potential duration of 118.3±15.2 ms in ventricular-like ES cell-derived cardiomyocytes selected using the Myl2 promoter. Taken together, these data demonstrate that iPS cell-derived myocyte lineage-committed cardiac progenitor cells are capable of further differentiating into fully mature and functional cardiomyocytes.

We have previously demonstrated that mouse ES-cell derived cardiomyocytes alone did not form functional cardiac biosynthetic tissue constructs and required additional input of cardiac fibroblasts; whereas a single cell source of ES-derived cardiac progenitors was sufficient to allow formation of constructs with advanced electromechanical properties [29]. Thus, for the purpose of this study we set out to determine whether mouse iPS cell-derived cardiac progenitors could be utilized to form functional tissue-like structures in a physiologically-relevant 3D configuration. We chose to utilize extracellular matrix materials that resemble granulation tissue (a rich mixture of fibrinogen, fibronectin, and collagen) [58], [59] that is formed from blood plasma components and fibroblast-secreted biomolecules. As early as 12–24 hours after myocardial infarction, early fibrin-rich granulation tissue begins to invade the infarct site, and forms a loose collection of pre-scar tissue for up to 2 weeks post-infarction [59], [60]. Granulation tissue is richly vascularized in the infarct border zone and has been shown to be a suitable environment for cell implantation therapies [60], [61].

We determined that iPS-derived cardiac progenitors in the 3D environment were able to differentiate into mature, striated cardiomyocytes with abundant electromechanical connections including adherens (Cdh2) and gap junctions (Gja1). We observed synchronous macroscopic contractions and uninterrupted action potential propagation throughout the construct, thus demonstrating that the differentiated cardiomyocytes had formed a well-integrated, functional tissue. We note that the circumferential pattern of gap junction expression around each cardiomyocyte and the relatively low action potential conduction velocity in these relatively pure cardiomyocyte tissues are characteristic of fetal-stage, immature myocardium [62], an observation consistent with our previous studies [29]. Furthermore, similar to previous cardiac tissue engineering studies, we observed that the cardiomyocytes formed a functional syncytium only in a relatively thin outer shell in the engineered tissues, suggesting that the survival and integration of these cells upon implantation will strongly depend on access to oxygen and nutrients. Overall, our findings suggest that the biosynthetic tissue constructs comprised of iPS-derived cardiac progenitors and composed of granulation-like tissue are appropriate for contributing to the formation of nascent vascularized myocardial tissue.

In conclusion, here we demonstrate that 1) mouse iPS-derived Nkx2-5(+) cardiac progenitors are multipotent and capable of differentiating into cardiomyocytes, smooth muscle, and endothelial cells. Moreover, progenitor cell selection enriches for a cKit(+) cell population and dual expression of either cKit/Flk1 or cKit/Sca-1 correlates with myocyte lineage commitment; 2) cardiac progenitor-descendent cardiomyocytes can reach an advanced stage of structural and functional maturity *in vitro*, and 3) when incorporated in a tissue engineered construct the cardiac progenitors are able to form well-integrated, functional tissues in a physiologically-relevant 3D environment. This strongly suggests that iPS cell-derived cardiac progenitors represent an excellent source for cell and tissue engineering therapies for heart disease as well as 3D culture platform for pharmacological screenings and drug development studies.

## Supporting Information

Figure S1 **Qualitative gene expression analysis (RT.PCR) for genes of pluripotency performed on populations on embryonic stem cells (ES D3, positive control), mouse embryonic fibroblasts (MEF, negative control), and induced pluripotent stem cells.** Primers designed specific for a region within the coding sequence are marked as “Ex/End” whereas primers designed specific for a region within the transcript but outside the coding region are marked as “End”.
(TIF)

Figure S2 **A**. Following plasmid DNA delivery by electroporation and antibiotic selection (neomycin), stably transfected iPS cells kept their morphological phenotype and continued to express pluripotency markers (Pou5f1, Fut4, and Nanog). **B**. Primers for gene expression analysis were designed specific for a region within the transcripted mRNA molecules of the *Pou5f1*, *Sox2*, *Klf4*, or *Myc* genes but outside the coding sequence in order to exclude expression from the lentivirally delivered transgenes. All gene expression levels were normalized against mouse embryonic fibroblasts using the ΔΔCt method. *, ** and ***indicate *p*<0.05, 0.01, 0.0001 computed using one-tailed Student’s t-test.
(TIF)

Figure S3 **Temporal qualitative gene expression analysis (RT.PCR) for pluripotency genes (** ***Pou5f1*** **,** ***Sox2*** **,** ***Nanog*** **), precardiac mesoderm genes (** ***T*** **,** ***Mesp1*** **), early cardiac transcription factors (** ***Nkx2-5*** **,** ***Gata4*** **,** ***Tbx5*** **,** ***Mef2c*** **,** ***Myocd*** **), and cardiomyocyte genes (** ***Nppa*** **,** ***Myl2*** **,** ***Myl7*** **,** ***Myh6*** **,** ***Myh7*** **,** ***Tnnt2*** **).**
(TIF)

Figure S4 **Monolayer cultures of neonatal rat ventricular myocytes were utilized as positive control to test the specificity and spatial organization of cardiomyocyte or smooth muscle proteins: Actn2, Tnnt2, Myh6, Myl2, Gja1, and Acta2.**
(TIF)

Table S1 **Primers used for quantitative or qualitative gene expression analysis.**
(XLSX)

Movie S1 **Cardiomyocytes within cultures of differentiating mouse iPS cells express the red fluorescent proteins and initiate spontaneous contraction within 7 days following initiation of differentiation.**
(MP4)

Movie S2 **Following puromycin selection (2 days) and enzymatic dissociation single cell suspensions of iPS-derived cardiac progenitors form compact monolayers and spontaneous contract.**
(MP4)

Movie S3 **Cells suspended in the hydrogel matrix within the PDMS molds initiate contractions.**
(MP4)

Movie S4 **Cells suspended in the hydrogel matrix within the PDMS molds initiate contractions.**
(MP4)

Movie S5 **Cells suspended in the hydrogel matrix within the PDMS molds initiate contractions.**
(MP4)

Movie S6 **Cells suspended in the hydrogel matrix within the PDMS molds initiate contractions.**
(MP4)

Movie S7 **Long-term culture of iPS-derived cardiac progenitors in the biosynthetic tissue constructs form mature cardiomyocytes, and compact the hydrogel matrix.**
(MP4)

Movie S8 **Calcium transient traversing a tissue patch from left to right as a result of action potential firing.** Red pixels represent a high intracellular calcium concentration, and blue pixels represent a low concentration.
(MP4)
