# Supplementary figures and images for: Induced Pluripotent Stem Cell-Derived Cardiac Progenitors Differentiate to Cardiomyocytes and Form Biosynthetic Tissues
Source: PLoS One. 2013 Jun 13;8(6):e65963. doi: 10.1371/journal.pone.0065963 (PMC3681781; doi:10.1371/journal.pone.0065963)

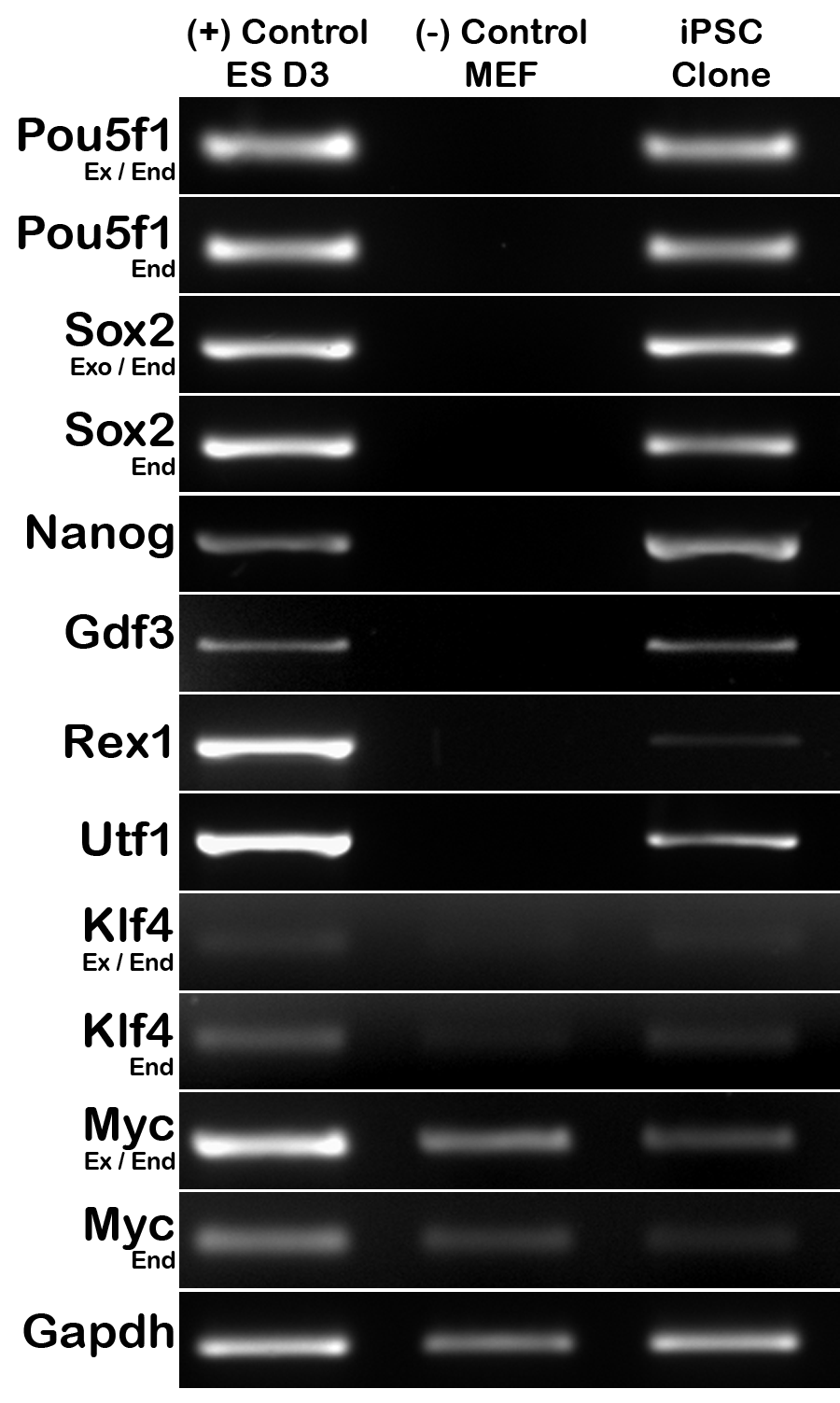

Supplement: Figure S1 — Qualitative gene expression analysis (RT.PCR) for genes of pluripotency performed on populations on embryonic stem cells (ES D3, positive control), mouse embryonic fibroblasts (MEF, negative control), and induced pluripotent stem cells. Primers designed specific for a region within the coding sequence are marked as “Ex/End” whereas primers designed specific for a region within the transcript but outside the coding region are marked as “End”. (TIF) [file pone.0065963.s001.tif]

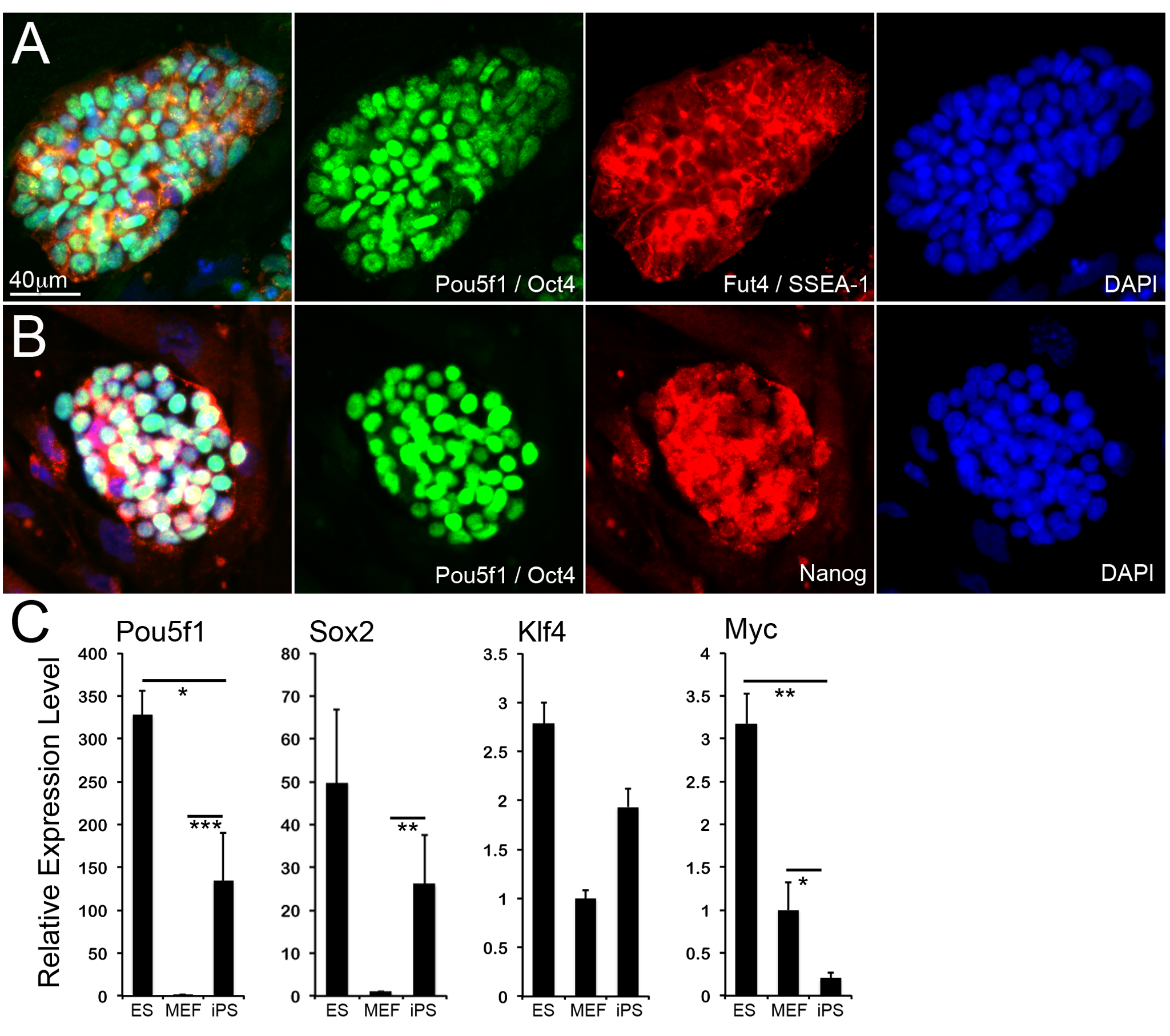

Supplement: Figure S2 — A. Following plasmid DNA delivery by electroporation and antibiotic selection (neomycin), stably transfected iPS cells kept their morphological phenotype and continued to express pluripotency markers (Pou5f1, Fut4, and Nanog). B. Primers for gene expression analysis were designed specific for a region within the transcripted mRNA molecules of the Pou5f1, Sox2, Klf4, or Myc genes but outside the coding sequence in order to exclude expression from the lentivirally delivered transgenes. All gene expression levels were normalized against mouse embryonic fibroblasts using the ΔΔCt method. *, ** and ***indicate p<0.05, 0.01, 0.0001 computed using one-tailed Student’s t-test. (TIF) [file pone.0065963.s002.tif]

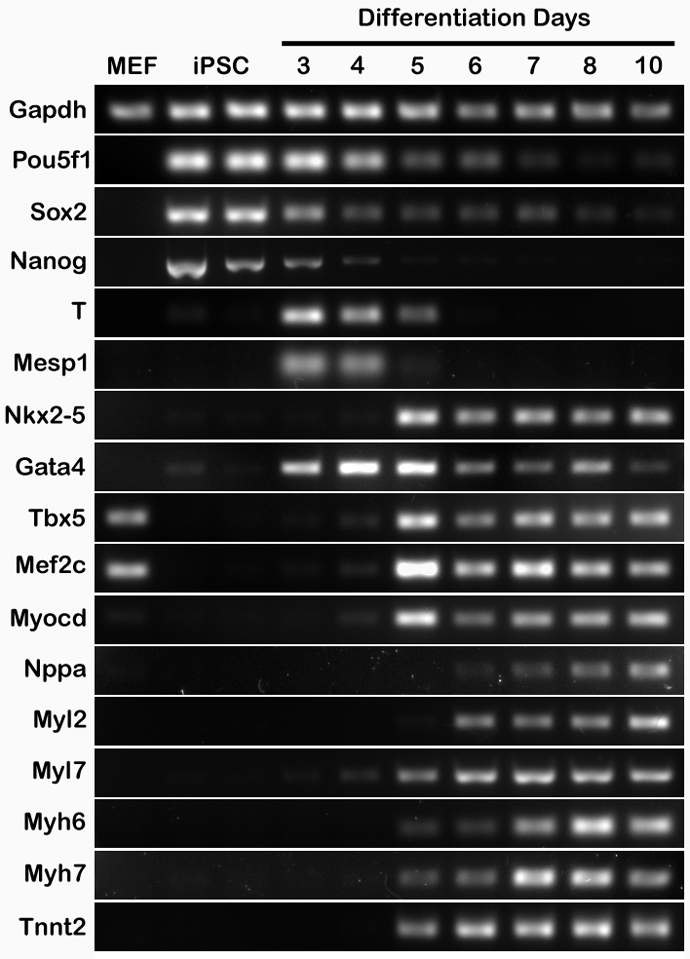

Supplement: Figure S3 — Temporal qualitative gene expression analysis (RT.PCR) for pluripotency genes ( Pou5f1 , Sox2 , Nanog ), precardiac mesoderm genes ( T , Mesp1 ), early cardiac transcription factors ( Nkx2-5 , Gata4 , Tbx5 , Mef2c , Myocd ), and cardiomyocyte genes ( Nppa , Myl2 , Myl7 , Myh6 , Myh7 , Tnnt2 ). (TIF) [file pone.0065963.s003.tif]

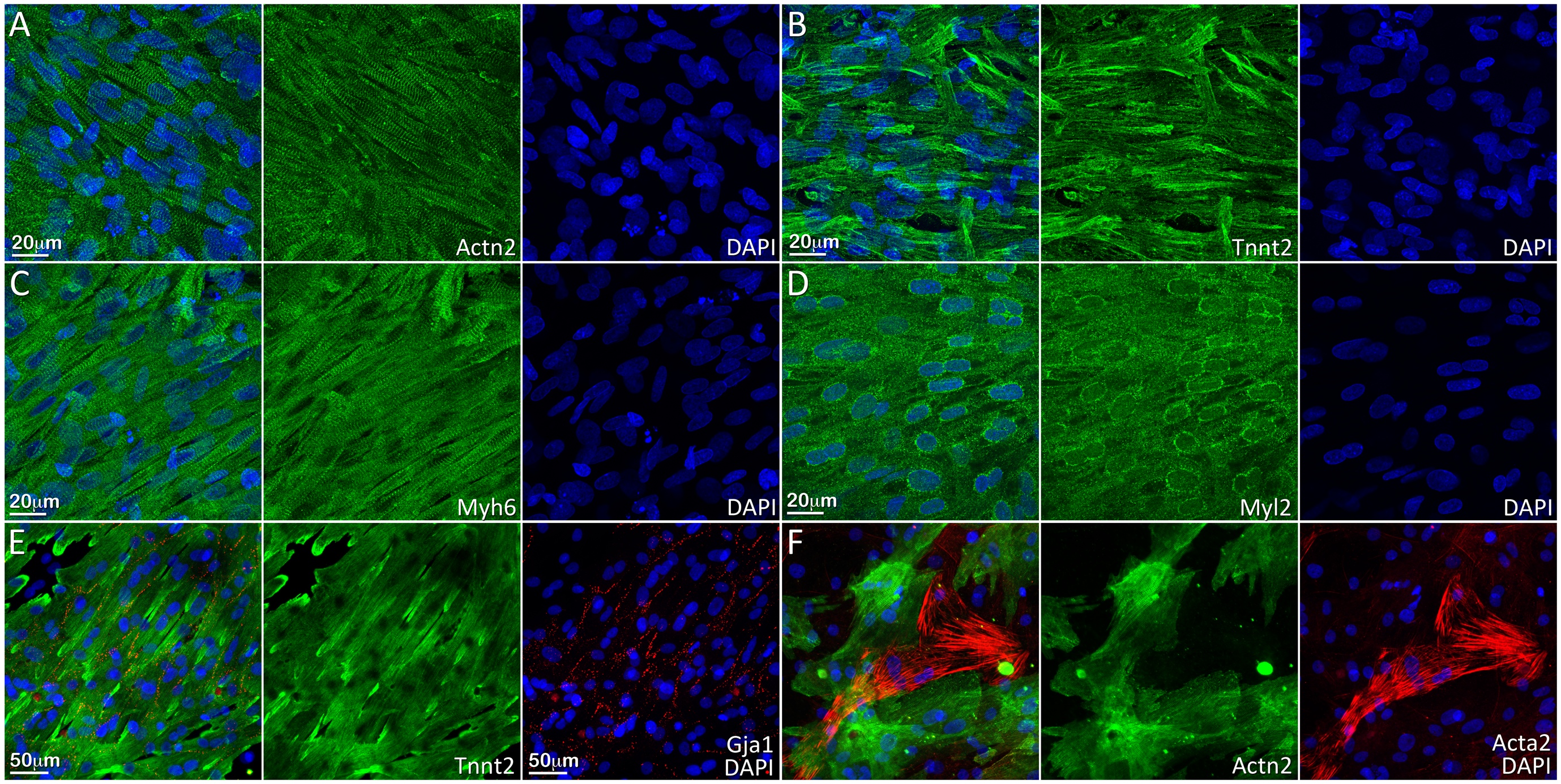

Supplement: Figure S4 — Monolayer cultures of neonatal rat ventricular myocytes were utilized as positive control to test the specificity and spatial organization of cardiomyocyte or smooth muscle proteins: Actn2, Tnnt2, Myh6, Myl2, Gja1, and Acta2. (TIF) [file pone.0065963.s004.tif]
